# Outcomes following second allogeneic stem cell transplant for disease relapse after T cell depleted transplant correlate with remission status and remission duration after the first transplant

**DOI:** 10.1186/s40164-018-0125-6

**Published:** 2019-01-03

**Authors:** Yun Fan, Andrew S. Artz, Koen van Besien, Wendy Stock, Richard A. Larson, Olatoyosi Odenike, Lucy A. Godley, Justin Kline, John M. Cunningham, James L. LaBelle, Michael R. Bishop, Hongtao Liu

**Affiliations:** 1 Department of Hematology, Beijing Hospital, Beijing, China; 2 Department of Medicine, Section of Hematology/Oncology, The University of Chicago Medical Center, 5841 S. Maryland, MC 2115, Chicago, IL 60637-1470 USA; 3 Division of Hematology/Oncology, Weill Cornell Medical College, New York, NY USA; 4 Department of Pediatrics, University of Chicago Comer Children’s Hospital, Chicago, IL USA

**Keywords:** Second allogeneic bone marrow transplantation, T cell depletion, Non-relapse mortality

## Abstract

**Background:**

Second allogeneic hematopoietic stem cell transplant (HCT) remains as an option for disease relapse after initial HCT.

**Methods:**

We analyzed retrospectively the outcomes of 65 consecutive patients who underwent a second HCT for disease relapse at the University of Chicago. Univariate and multivariate analysis were conducted, and a scoring system was generated to select the patients who would benefit second HCT.

**Results:**

All except four patients received T cell depleted (TCD) first HCT. The majority of patients had AML (n = 47) and high risk MDS (n = 5). The median age at second HCT was 45 years (11–73). 13 patients (20%) achieved CR before second HCT. 98% (n = 64) and 72% (n = 47) patients achieved neutrophil and platelet engraftment at a median interval of 10 and 18 days, respectively, following the second HCT. With a median follow up of 23 (5.5–140) months for survivors after second HCT, the estimated 2 years PFS was 17.5% and the 2 years OS was 22.6%. The day 100 cumulative incidence of non-relapse mortality rate was 23.6%, and the cumulative incidence of aGVHD and cGVHD were 16.9% and 7.7% respectively at 1 year after second HCT. In univariate analysis, patients with remission duration after first HCT of > 12 months and those in CR before second HCT had significantly better PFS and OS. A scoring system using disease status before second HCT (CR = 0 vs. non-CR = 1), and remission duration after first HCT (< 6 = 2, 6–12 = 1 and > 12 months = 0) was generated as an approach to classify patients into different risk categories in the purpose to provide guidance to the transplant physician to inform the outcomes to potential patients undergoing 2nd HCT. A score of < 2 (n = 26) identified a group with PFS and OS of 31.6% and 36.2% at 2 years after second HCT.

**Conclusion:**

In conclusion, second HCT is a viable option for disease relapse after TCD HCT for patients entering second HCT in remission and/or remission duration > 12 months after first HCT with acceptable rates of GVHD and donor engraftment.

## Introduction

HCT is the only curative treatment for many high-risk hematologic diseases, including AML, and MDS. The numbers of HCT are steadily increasing worldwide, with improving outcomes as judged by lower treatment-related mortality. However, relapse after HCT remains the major cause for treatment failure. In most situations, treatment options at relapse after HCT are very limited with unacceptably high rates of disease relapse. The treatment options for disease relapse after HCT include withdrawal of immune suppression, chemotherapy, second allogeneic transplant, cytokine and adoptive cell therapy and donor lymphocyte infusion [1].

Second HCT has been demonstrated repeatedly to provide benefit, albeit in a small subset of patients [2–5], with larger more recent retrospective studies confirming this observation [6–8]. In addition, a recent retrospective registry study performed by the Acute Leukemia Working Party of EBMT reported the outcomes of adults with relapsed AML after RIC HCT [9]. Long-term survival was only achieved in patients who achieved CR by cyto-reductive therapy, followed either by donor lymphocyte infusion or second HCT as consolidation [9].

T-cell depletion is an approach that enhances procedure tolerability by reducing acute and chronic GVHD related morbidity and mortality. We have employed in vivo T-cell depletion with alemtuzumab for over a decade at the University of Chicago and confirmed lower rates of acute and chronic GVHD with similar overall survival to T cell repleted HCT [10]. Although relapse remains problematic after transplant [11], particularly for those with active disease at time of HCT, there has been concern that T-cell depletion might further increase the relapse rate. Though registry studies suggest an increased rate of disease recurrence after T-depleted transplantation [12], our own comparative analysis of alemtuzumab vs non-alemtuzumab based conditioning did not show an increased relapse incidence [10]. Neither did another study comparing CD34 selection with conventional GVHD prophylaxis [13, 14].

In this study, we analyzed outcomes in patients, many of whom received an initial T cell-depleted transplant, who then received second HCT at our center for relapsed disease. It is a retrospective single center analysis.

## Patients and methods

Sixty-five consecutive patients who underwent second HCT for disease relapse at University of Chicago were retrospectively analyzed. This study included all the patients who received second HCT at our center without selection, and these patients who were able to move to second HCT could be well-selected since second HCT was only one of the options to treat disease relapse after HCT. Patient characteristics were listed in Table 1. All except 4 patients received T cell depleted (TCD) HCT as first HCT. The majority of patients (71%) received RIC during first HCT. As a strategy to prevent further relapse, the majority of patients (68%, n = 44) received myeloablative conditioning regimens for their second transplant. 18 patients received Fludarabine (Flu)-Busulfan (Bu)-alemtuzumab (campath), 7 patients received TBI-based regimens and two patients received Bu/cyclophosphamide (CTX). Twenty-one patients received reduced intensity conditioning including 5 patients received Flu-Mel-alemtuzumab, 7 patients received Clofarabine (Clo)-Mel-Campath and 7 patients received Flu-Mel-ATG. Tacrolimus and MMF were mainly used as GVHD prophylaxis. This study included a heterogeneous patient population, while the majority of the patients had AML/MDS and underwent RIC conditioning for second HCT.

**Table 1:** Characteristics of the patients

Variable	Total
Total patients	65
Median Age (range)	45 (11–73)
Male/female	48/17
Diagnosis	
AML	47
MDS	5
Lymphoma	6
CML	2
Other leukemia	5
Disease status before second HCT	
Remission	13
Not in remission	52
Donor status	
MRD	33
MUD	21
Haplo-cord	11
Conditioning regimen for first HCT	
Myeloablative	19
RIC	46
Conditioning regimen for second HCT	
Myeloablative	44
Flu-Bu-Alemtuzumab	18
TBI-based	7
Bu/Cy	7
RIC	21
Flu-mel-Alemtuzumab	5
Clo-mel-Alemtuzumab	7
Flu-mel-ATG	7

*MRD* matched related donor, *MUD* matched unrelated donor, *MDS* myelodysplastic syndrome

### Endpoints

The primary endpoints of the retrospective analysis were OS, NRM, and progression-free survival (PFS). The analysis included OS, PFS, relapse, and NRM.

### Statistical analysis

Patient and transplantation characteristics were analyzed using descriptive statistics. NRM estimates were calculated using cumulative incidence with relapse as the competing event. The estimated relapse was calculated using cumulative incidence with NRM as competing event. The estimate of aGVHD/cGVHD of second HCT was calculated using cumulative curves in the competing risk model. The estimate of time to engraftment of ANC and PLT was calculated using life tables. Successful engraftment was set as the positive event. The probabilities of OS and PFS were analyzed using Kaplan–Meier plotting and its univariate analysis. PFS was defined as time to relapse or death from any cause. OS and PFS were calculated from the date of second transplant. The p value was set at < .05 for statistical significance. In terms of multivariate analysis, Cox regression model was applied in which the OS and PFS was dependent variable respectively. Statistical analyses were performed with SPSS software.

## Results

### Patient characteristics

A total of 65 consecutive patients were included (Table 1). Forty-eight patients were male and 17 were female. The median age at second transplantation was 45 years (range 11 to 73 years). Underlying diagnoses were as follows: 47 patients were diagnosed with AML (72%) and 5 (8%) patients were diagnosed with MDS. Remission duration after first HCT was calculated from the date of first HCT to the date of disease relapse after first HCT. All the 65 patients had disease relapse after first HCT. All the relapsed patients after first HCT received disease control treatment prior to second HCT, but 52 (80%) patients still had active disease while 13 (20%) patients were in complete remission (CR) after treatment prior to second HCT. The donor for the second HCT was MRD in 33 patients, MUD in 21 patients, and haplo-cord in 11 patients. Forty-four patients (68%) received myeloablative conditioning (MAC), and 21 (32%) received a reduced intensity condition HCT. Most of the patients (73.8%, 48/65) still received T cell depleted HCT either with Campath for matched donor or ATG for haplo-cord donor for the second HCT. Further information of patient characteristics can be found in Table 1. More than half (58%) patient had duration of remission after first HCT that persisted for greater than 6 months prior to second HCT (Table 2), 75% patients were able to move to second HCT within 6 month after disease relapse from the first HCT. In these selected patients for the second HCT, it was not surprised that only six patients developed Grade II aGVHD, and only 2 patients had mild cGVHD after first HCT and before second HCT.

**Table 2:** Information of patients before the second HCT

Characteristics	No (%)
Duration of remission after first HCT (months)	
< 6	27 (42%)
≥ 6–12	15 (23%)
≥ 12	23 (35%)
Time from relapse to second HCT (months)	
< 6	49 (75%)
≥ 6–12	12 (18%)
≥ 12	4 (6%)

### Hematopoietic recovery after second HCT

Following the second HCT, 64 patients (98%) achieved engraftment of neutrophil and 47 patients (72%) achieved engraftment of platelet with median times of 10 days (8–55 days) and 18 days (8–146 days) respectively (Table 3). The cumulative incidence of day 28 neutrophil and platelet engraftment was 98% and 73% respectively (Table 3). Donor engraftment analysis were routinely done in all the patients, and most of patients had full donor chimerism at day 30 after second HCT, but the data was not captured in this analysis as part of hematopoietic recovery after second HCT.

**Table 3:** Transplant outcomes after second HCT

Characteristics	No (%)
Neutrophil recovery	
Neutrophil engraftment by day 28	98%
Median (day)	10 (8–55)
Platelet recovery	
Platelet engraftment by day 28	73%
Median (day)	18 (8–146)
NRM	
Day 100	23.6%
1 year	36.9%
aGVDH Grade II–IV at 1 year	16.9%
cGVHD at 1 year	7.7%
Relapse at 1 years	33.8%
1 year PFS	29.3%
1 year OS	33.3%
Death	47
Relapse	22 (47%)
Infection	11 (23%)
GVHD	6 (13%)
Other	8 (17%)

### Survival, relapse, NRM, and GVHD after second HCT

**Fig. 1:**
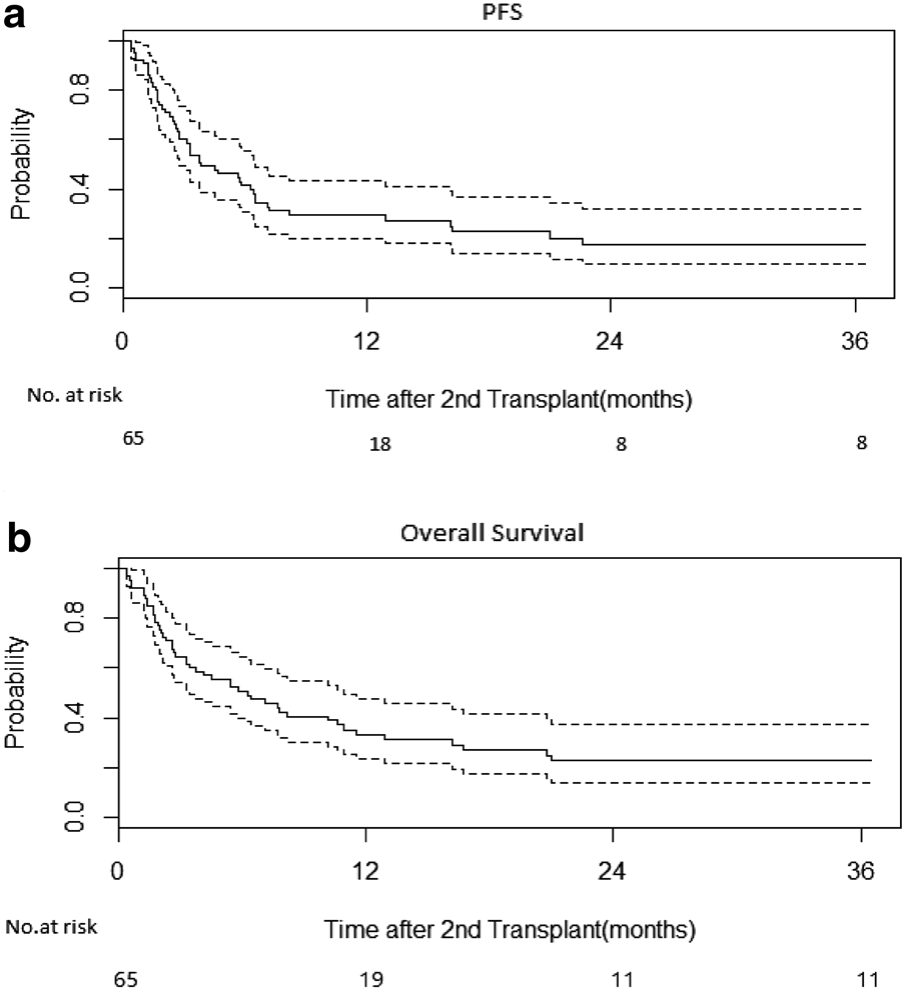
Long term survival could be achieved in patients after second HCT. The estimated 2 years PFS and OS was 17.5% and 22.6% respectively

With a median follow-up of surviving patients of 695 days (range 166 to 4191 days), PFS and OS were 29.3% and 33.3% at 1 year respectively (Fig. 1), and both curves stayed flat after 2 years follow-up, indicating that very few patients were at risk of disease relapse more than 2 years after second HCT. The cumulative incidence of relapse was 36.9% at 1 year. The cumulative incidence of non-relapse mortality (NRM) was 23.6% at day 100, and 33.8% at 1 year after the second HCT (Table 3). The cumulative incidence of acute GVHD was 16.9%, and of chronic GVHD was 7.7% at 1 year post-transplant (Table 3). There were 47 deaths at the end of follow-up. The most common cause of death was disease relapse in 22 patients (47%), followed by infection in 11 patients and GVHD in 6 patients (Table 3).

Univariate analysis was utilized as an approach in identifying the factors affecting the PFS and OS after second HCT. Factors evaluated included pre-transplant patient characteristics, conditioning regimen, donor options etc. In our analysis with limited power due to small patient number, there was no association of PFS and OS with donor type(MRD vs MUD vs Haplo-cord),performance status (≥ 90 vs < 90), HCT-CI(≥ 3 vs < 3), conditioning intensity (Myeloablative vs RIC), development of GVHD after second HCT or not, patient age (≤ 60 vs. > 60), or use of alemtuzumab as part of conditioning regimen for the second HCT (data not shown).

**Fig. 2:**
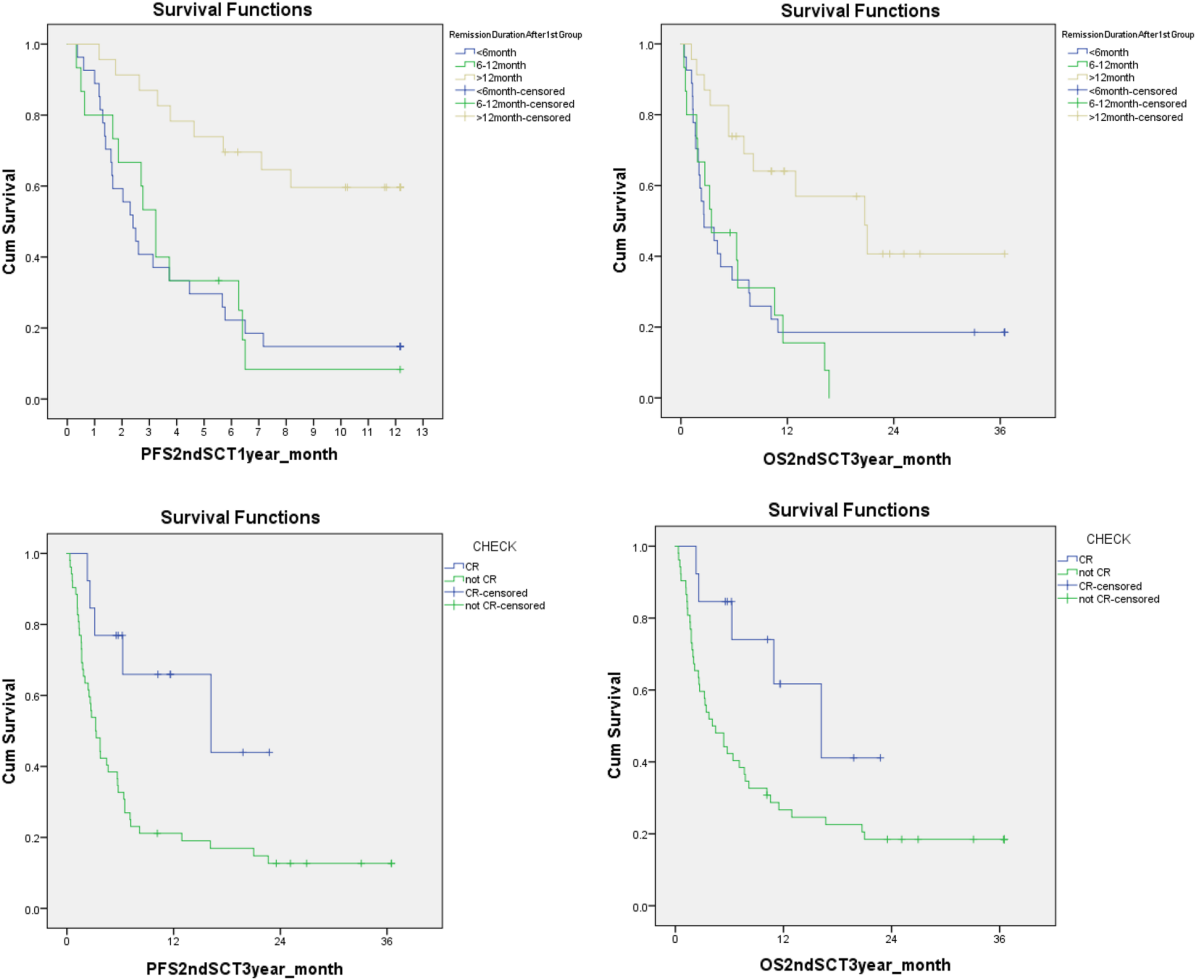
Long duration of remission after first HCT and disease remission prior to second HCT predicted better survival significantly after second HCT. Patients whose remission duration > 12 months after first HCT had significant better outcome (top panels), as well as the patients who entered complete remission prior to second HCT (bottom panels)

In contrast, univariate analysis demonstrated that patients with remission duration after first HCT of > 12 months or being in CR prior to the second HCT was associated significantly with better PFS and OS respectively. The difference of PFS and OS stratified by these two variables was very striking (Fig. 2).

In addition, Cox regression model was applied for multivariate analysis in which the OS and PFS was dependent variable respectively. According to the univariate analysis, remission duration after first HCT and being in CR prior to the second HCT or not was input in the model. In the PFS model, remission duration and being in CR prior to the second HCT or not were risk factors. The patients whose remission duration after first HCT < 12 months had high risk of relapse or death than those whose duration > 12 months, with a hazard ratio (HR) of 1.54. Patients who were not in CR prior to second HCT had high risk of relapse or death than those who were in CR, with a HR of 2.48. In the OS model, only being in CR prior to the second HCT or not had been kept in the Cox equation. Patients who were not in CR prior to second HCT had high risk of death than those in CR, with a HR of 31.80.

**Fig. 3:**
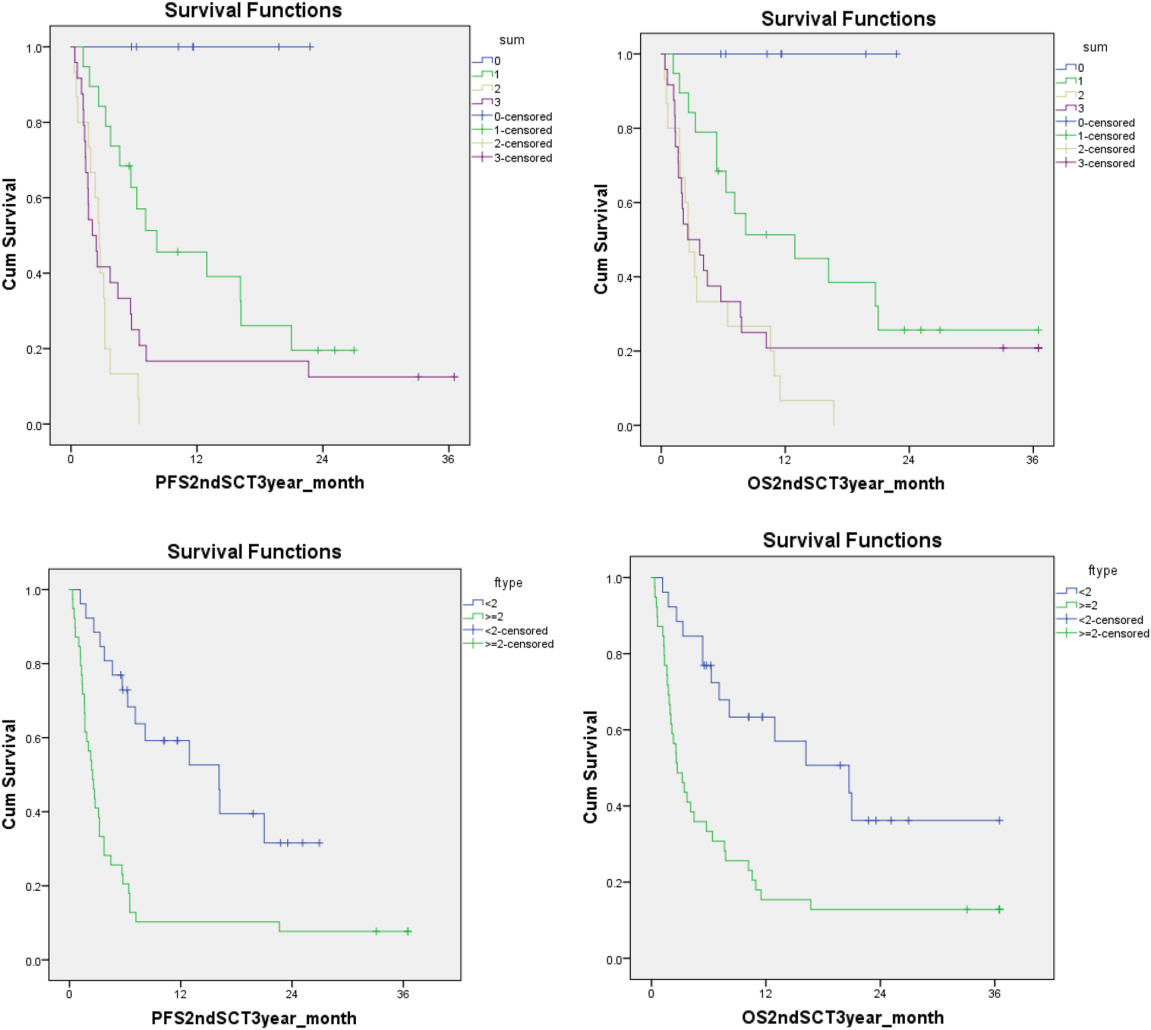
A scoring system using disease status before second HCT and remission duration after first HCT can predict survival outcome after second HCT. Disease status before second HCT (0: CR vs. 1: non-CR) and remission duration after first HCT (0: > 12 months; 1: 6 to 12 months; 2: < 6 months) could be used to predict the outcomes after second HCT. The outcomes of the patients with a score < 2 were significant better than those who ≥ 2, which selected out the patients who could benefit most from second HCT

In the purpose to provide guidance to the transplant physician to inform the outcomes to the potential patients undergoing 2nd HCT, we built a scoring system using the two variables which were statistically significant in univariable analysis: remission status prior to second HCT and remission duration after first HCT (Table 4); The scoring system could separate the PFS and OS effectively (Fig. 3, top panel); Summarizing the use of this scoring system, patients who scored < 2 demonstrated statistically significant better 2 years PFS of 31.6% and OS of 36.2% comparing to the patients who scored ≥ 2 at 7.7% and 12.8% as 2 years PFS and OS respectively (Fig. 3, low panel). Thus, patients who score < 2 on the scoring system might be considered for second HCT given the enhanced possibility for long-term survival. In order to make this score system valid to apply to other transplant centers, it will need to be validated using much larger patient population, which is not possible at this time. This scoring system has been routinely used at our transplant center to guide the decision to move forward to second HCT, and to inform the potential patients the outcome after second HCT to obtain informed consent for second HCT.

**Table 4:** A scoring system using the remission status prior to second HCT, and remission duration after first HCT

Score	CR or not Prior to second HCT	Remission duration after first HCT (months)
0	CR	> 12
1	Not CR	6–12
2		< 6

## Discussion

Relapse remains the leading cause of treatment failure in hematologic malignancies after allogeneic stem cell transplant. Treatment options for disease relapse after HCT remains limited, and long term survival even with new agents is disappointing. Although there is significant mortality, second HCT has been demonstrated to provide enhanced long term survival in well-selected patients [6–8, 15]. Our center has been using in vivo T cell depletion with alemtuzumab as part of the conditioning regimen. Our single center retrospective study presented the analysis of the second HCT outcomes for disease relapse after T cell depleted HCT in patient with hematologic malignancies. As we are aware, it is the largest retrospective study focusing T cell depleted HCT to determine the outcomes after second HCT.

Limited by relatively small patient numbers, a heterogeneous population with different disease diagnosis, disease and donor status and conditioning regimens, our results showed that donor type (MRD vs. MUD vs. haplo-cord), conditioning intensity (RIC vs. myeloablative), and patient age had no influence on PFS and OS. We could not exclude that these variables might have effect in a much large analysis. While our analysis could not confirm the detrimental effects on survival from T-cell depletion with alemtuzumab, avoidance of T cell depletion using alemtuzumab especially in patients receiving second matched related donor HCT had been recommended in our center in the recent years of clinical practice. In the univariate analysis, patients with remission duration after first HCT (> 12 months) or CR status before second HCT had significantly better PFS and OS. These observations suggested that the remission duration and CR status may serve as positive predictive prognostic factors for survival after second HCT at least in our patient population undergoing T cell depleted HCT. The scoring system we devised using disease status before second HCT (CR vs. non-CR), and remission duration after first HCT (< 6, 6–12 and ≥ 12 months) (Table 4) could be utilized to identify patients who might benefit second HCT effectively (Fig. 3). Patients scored < 2 on the scoring system had enhanced 2 years PFS and OS, and second HCT might be considered in these well selected population.

Our study is consistent with several recently published retrospective studies demonstrating that the duration of first HCT remission and diseases status prior to second allo-ST were important factors in determining the outcome after second HCT [6–8, 15]. Specifically, Ruutu et al. retrospectively analyzed the outcome of predictive factor after second HCT using data from EBMT; they demonstrated that an advanced stage of disease predicted poor outcome; a longer remission after the first transplantation was a favorable predictive factor for survival; and the patients with an interval of more than 1 year between the transplantations had a clearly better survival than those with a shorter interval [8]. Our data demonstrated that for patients who relapsed after T-cell depleted HCT, second HCT remains a viable option for selected patients. Similar to the observation by Ruutu et al. the best outcomes were achieved in patients who relapsed more than 12 months after the first HCT, and disease in remission prior to second HCT (Fig. 3) in our T cell depleted first HCT patients. Our results are also consistent with the observations of Schmid et al. in relapsed AML patients after RIC HCT. In their analysis, only patients who could enter CR after chemotherapy induction followed by cellular therapies, either donor lymphocyte infusion or second HCT, were long-term survivors [9].

In addition, according to the univariate analysis results, a scoring system using remission status and remission duration after first HCT was generated; and patients who scored < 2 on the scoring system demonstrated decent 2 years PFS of 31.6% and OS of 36.2% comparing to the patients who scored ≥ 2 at 7.7% and 12.8% of 2 years PFS and OS respectively. Thus, patients who score < 2 on the score system should be considered for second HCT for long-term survival.

Due to the very high 1 year NRM at 33.8% and high relapse rate at 36.9% at 1 year for the whole population, and 2 years PFS of only 7.7% for patients scored ≥ 2 in the scoring system, patients who scored ≥ 2 on the scoring system might not be offered second HCT, while clinical trial using targeted therapies or novel immunotherapies including antibody-based therapy or CAR-T cell therapy if available should be considered other than second HCT. The ongoing development of novel agents and treatment modalities may help to place more patients who relapsed after HCT into a further complete remission or decent disease control allowing a new bridge to second HCT.

Not surprisingly, disease relapse remains the major cause of mortality even after second HCT, and it contributed to 47% death (Table 3) after second HCT in our analysis. The strategies to prevent disease relapse after second HCT will not differ significantly from those available prior to the first HCT: (1) to achieve deeper minimal residual disease negative remission with novel agents since minimal residual disease prior to HCT predicts disease relapse [16–18]; (2) novel conditioning approaches to eliminate residual disease without increase of toxicities; (3) early intervention after HCT to prevent disease relapse, including maintenance treatment or prophylactic intervention, like prophylactic donor lymphocyte infusion or immunotherapy. In this regard, we do have ongoing clinical trial to incorporate Intensity Modulated Total Marrow Irradiation (IM-TMI) with fludarabine and melphalan as conditioning regimen for patients undergoing second HCT (NCT02333162) with the hope to eliminate residual disease prior to second HCT to prevent disease relapse after second HCT.

Our analysis had several limitations. The sample size precludes subset analysis or robust multivariate analysis. The predominance of T-cell depletion for first and second HCT may reduce generalizability. Finally, as with most series, patients undergoing second HCT were likely a selected subset of patients who relapsed.

## Conclusion

In conclusion, second HCT is a viable option for disease relapse after T cell depleted HCT with acceptable GVHD and good engraftment for those entering transplant in remission with remission duration > 12 months after first HCT.

## Abbreviations

AE: adverse event
AML: acute myeloid leukemia
ANC: absolute neutrophil count
ECOG: Eastern Cooperative Oncology Group
G-CSF: granulocyte-colony stimulating factor
MDS: myelodysplastic syndromes
NE: not estimable
OS: overall survival
RBC: red blood cells
PFS: progression free survival
MRD: matched related donor
MUD: matched unrelated donor
Haplo-Cord: haploidentical-cord
HCT: allogeneic stem cell transplantation
GVHD: graft versus host disease
CR: complete remission
HCT-CI: hematopoietic cell transplantation-comorbidity index
